# C-Reactive Protein in Saliva as a Non-Invasive Marker of Metabolic Syndrome: A Systematic Review and Meta-Analysis

**DOI:** 10.3390/life16030403

**Published:** 2026-03-02

**Authors:** Mohammad Khalfan, Yash Brahmbhatt, Sarah Pagni, Ripple Garg, Ahmad A. Alkandari, Abrar Alkhesaili, Nouf Alsheredah, Nawal AlDhafeeri, Hawra Baroon, Woroud Al-Sulimmani, Shaikha Almatrouk, Fahad Alali, Hend Alqaderi

**Affiliations:** 1 College of Medical, Veterinary & Life Sciences (MVLS), Dental School, University of Glasgow, 378 Sauchiehall Street, Glasgow G2 3JZ, UK; 2508013k@student.gla.ac.uk; 2 School of Dental Medicine, Tufts University, Boston, MA 02111, USAsarah.pagni@tufts.edu (S.P.); ripple.garg@tufts.edu (R.G.); 3 Kuwait Ministry of Health, Kuwait City 15462, Kuwait; dr.ahmadaalkandari@gmail.com (A.A.A.); abraralkhesili@gmail.com (A.A.); noufalsheredah@gmail.com (N.A.); nawals.aldhafeeri@gmail.com (N.A.); hawra.baroon@gmail.com (H.B.); woroudnm@hotmail.com (W.A.-S.); shaikhah14bsk@gmail.com (S.A.); drfahadba950@gmail.com (F.A.); 4 Dasman Diabetes Institute, Kuwait City 15462, Kuwait; 5 Tufts Institute of Artificial Intelligence, Tufts University, Medford, MA 02111, USA

**Keywords:** salivary C-reactive protein, CRP, metabolic syndrome, salivary biomarkers, systemic inflammation, cardiometabolic risk, meta-analysis

## Abstract

Metabolic syndrome (MetS) is a cluster of conditions that rely on low-grade systemic inflammation and increase the risk of cardiovascular disease, type 2 diabetes, and stroke. C-reactive protein (CRP) has attracted growing interest in saliva as a non-invasive alternative to serum CRP testing, though existing evidence remains inconsistent. This systematic review and meta-analysis evaluated the association between salivary CRP levels and MetS and examined the consistency of findings across populations and methodological approaches. PubMed, the Cochrane Library, and Web of Science were searched up to December 2024 following PRISMA 2020 guidelines. Nineteen studies involving 3265 participants with and without MetS were included. Random-effects meta-analysis demonstrated higher salivary CRP levels in individuals with MetS compared with controls (SMD = 1.02; 95% CI −0.23 to 1.81), with substantial heterogeneity (I^2^ = 98.91%), reflecting variation in population characteristics and saliva collection protocols. Funnel plot assessment did not indicate publication bias. Despite considerable heterogeneity, pooled estimates suggest that individuals with MetS have higher levels of salivary CRP. Longitudinal studies employing standardized methodologies are required before its clinical implementation can be considered.

## 1. Introduction

Metabolic syndrome (MetS) has emerged as a significant global health challenge, characterized by a constellation of interconnected metabolic abnormalities, including central obesity, elevated blood pressure, dyslipidemia, insulin resistance, and impaired glucose regulation [1]. Together, these risk factors significantly elevate the likelihood of developing type 2 diabetes (T2D) and cardiovascular diseases (CVDs), the two leading causes of global morbidity and mortality [2]. Alarmingly, current estimates suggest that nearly one-quarter of the world’s adult population is affected by MetS, with prevalence continuing to rise in parallel with the global obesity epidemic and sedentary lifestyles [3,4].

Although MetS is not classified as a disease in itself, it functions as a potent indicator of underlying pathophysiological dysregulation [5]. The clustering of its components reflects a shared basis in chronic low-grade systemic inflammation and insulin resistance [6]. One of the challenges with MetS is its insidious progression [7]. Often asymptomatic in its early stages [2], it may go undetected until serious complications such as myocardial infarction [8], stroke [9], or T2D manifest [10]. This silent nature underscores the need for early identification and proactive management of individuals at risk [11].

A growing body of evidence indicates that elevated circulating inflammatory mediators contribute to endothelial dysfunction and accelerate the development of MetS and its individual components [6,11]. Several inflammatory biomarkers have been implicated in this inflammatory process, including interleukin (IL)-6, IL-8, IL-10, leptin, insulin, vascular endothelial growth factor (VEGF), monocyte chemoattractant protein-1 (MCP-1), and C-reactive protein (CRP) [6,12,13,14]. Among the wide range of candidate salivary biomarkers, CRP is one of the most clinically relevant inflammatory markers associated with a wide range of inflammatory conditions, specifically metabolic conditions [15,16]. Serum CRP reflects hepatic acute-phase responses to cytokines such as interleukin-6 (IL-6) [15,17]. The onset and severity of MetS are frequently linked to elevated CRP [18,19], indicating its value in risk stratification and monitoring disease progression [20,21,22,23]. Importantly, CRP is also detectable in saliva [24,25,26,27,28,29]. Its presence in the oral cavity is thought to arise from passive diffusion and ultrafiltration from the bloodstream, as well as transudation through gingival crevicular fluid [25,26,28]. These mechanisms are accentuated during systemic inflammation [26]. Because salivary CRP largely reflects serum CRP, salivary concentrations have been shown to correlate with systemic levels [25,26,27,30,31,32,33,34], supporting the premise that saliva may serve as a practical, non-invasive medium for assessing the inflammatory burden characteristic of MetS.

Despite its clinical utility, measuring CRP in serum presents practical limitations, particularly in large-scale screening contexts or among populations where venipuncture is not feasible [17,24]. Blood collection is invasive, potentially distressing, and may require trained personnel, sterile equipment, and controlled conditions [17]—all of which are barriers in low-resource settings or when high-frequency monitoring is necessary. This has led to growing interest in saliva as a diagnostic fluid [28,29,30,31,32]. Saliva offers a convenient, non-invasive, and cost-effective alternative to blood, with the additional advantage of easy and repeated sampling [27,28,29,30,31,32,33]. The use of saliva for diagnostic purposes is expanding across several domains, including infectious diseases, hormonal disorders, and cardiovascular risk assessment [29,30,31,32,33,34]. Importantly, emerging evidence suggests that salivary CRP levels strongly correlate with serum CRP concentrations [25,26,27,35], indicating that salivary CRP may reflect systemic inflammatory status and thus hold potential as a detecting biomarker for MetS.

Despite the growing number of studies investigating salivary CRP, the literature remains fragmented and inconclusive [35,36,37,38]. Variability in population characteristics, saliva collection protocols, CRP assay methods, and diagnostic criteria has contributed to inconsistent findings. Although some studies have found strong associations between salivary CRP and cardiometabolic risk phenotypes [39,40,41], the consistency of salivary CRP in detecting early metabolic dysfunction compared to its serum counterpart remains inconsistent [42,43,44,45,46,47,48,49,50,51,52,53,54,55,56,57,58,59]. Given this gap in knowledge, there is a compelling need to systematically evaluate the existing evidence. A comprehensive review of the current research is necessary to determine whether salivary CRP can be considered a reliable, non-invasive alternative to serum-based CRP testing in identifying individuals at risk for MetS.

Therefore, this systematic review and meta-analysis represents the first quantitative synthesis to assess the association between salivary CRP levels and metabolic syndrome-related cardiometabolic phenotypes. By pooling data across diverse populations and study designs, this study aims to (i) estimate the magnitude of difference in salivary CRP between individuals with and without metabolic dysfunction, and (ii) explore sources of heterogeneity related to assay methodology and diagnostic definitions. Clarifying this evidence base may inform future screening strategies and support the development of accessible, saliva-based approaches for metabolic risk assessment, particularly in community and resource-limited settings.

## 2. Materials and Methods

### 2.1. Search Strategy

**Figure 1:**
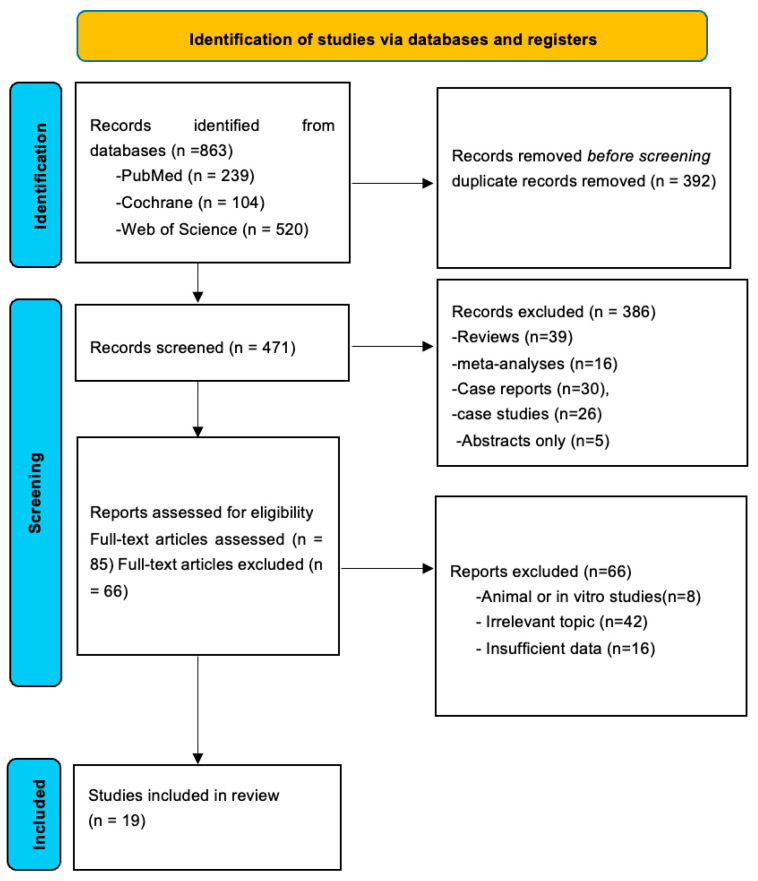
PRISMA flow diagram of study selection for the meta-analysis.

This systematic review and meta-analysis was conducted in accordance with the Preferred Reporting Items for Systematic Reviews and Meta-Analyses (PRISMA) 2020 guidelines [60]. A structured literature search was performed in three electronic databases, PubMed, Cochrane Library, and Web of Science, covering all records from inception to December 2024. A PRISMA flowchart of the selection process is shown in Figure 1. Supplementary Materials include the complete search strategy and a list of the (*n* = 66) excluded reports.

The PECO (Population, Exposure, Control, Outcome) framework guided study selection. The target population included individuals of all age groups, genders and geographic locations who have metabolic syndrome (MetS). Accepted diagnostic criteria for MetS included NCEP ATP III, IDF, or WHO guidelines [5,39], as shown in Table 1. The exposure of interest was salivary C-reactive protein (CRP) levels. The control group consisted of individuals without MetS or any of its individual components (central obesity, dysglycemia, hypertension, T2D or dyslipidemia). The outcome of interest was one or more MetS risk factors including central obesity (waist circumference, BMI), hypertension, dyslipidemia (high LDL or low HDL) and CVD.

Search terms included combinations of “saliva,” “salivary,” “C-reactive protein,” “CRP,” and metabolic-syndrome–related terms such as “metabolic syndrome,” “MetS,” “insulin resistance,” “cardiometabolic risk,” “Obesity” “dyslipidemia,” “hyperglycemia,” and “hypertension.” Terms relating only to single metabolic abnormalities (e.g., “obesity,” “type 2 diabetes”) were not used as standalone identifiers for MetS. These terms were used with combinations of “OR” and “And”. No language restrictions were applied. After duplicate removal, two reviewers independently screened titles and abstracts before conducting full-text assessment (Figure 1).

### 2.2. Eligibility Criteria

Inclusion and Exclusion Criteria

Studies were included if they met the following criteria:

- Original, peer-reviewed human research.
- Quantitative measurement of salivary C-reactive protein (CRP).
- Participants diagnosed with metabolic syndrome (MetS) according to established criteria (NCEP ATP III, IDF, or WHO) or with cardiometabolic conditions related to MetS, including obesity or overweight states, hyperglycemia or type 2 diabetes, hypertension, or cardiovascular risk phenotypes.
- Inclusion of a comparator group consisting of individuals without MetS or any of its individual components.
- Sufficient data available to extract or calculate mean and variance for salivary CRP.

Exclusion criteria were:

- Non-human, in vitro, or ex vivo studies.
- No quantitative salivary CRP measurement.
- No appropriate comparison group.
- Studies not related to metabolic dysfunction such as inflammation from infection, autoimmune disease, or oral pathology.
- Publication types such as reviews, systematic reviews, meta-analyses, case reports, case series, conference abstracts, editorials, and commentaries.
- Duplicate publications or overlapping datasets (the most complete dataset was retained).

When data were missing or unclear, study authors were contacted. Two of the three contacted authors provided the required information; one study was excluded due to non-response.

### 2.3. Data Extraction and Quality Assessment

Study quality was assessed using the Newcastle–Ottawa Scale (NOS) for observational studies [40]. NOS assesses the quality of non-randomized studies across three domains: selection of study groups, comparability of groups, and ascertainment of the outcome, using a star-grading system, with a maximum of nine stars indicating the highest quality [40,41,42]. Studies scoring 1–3 were classified as low quality, 4–6 as moderate, and 7–9 as high quality [42]. Discrepancies in extracted data and quality assessment were resolved through consensus.

### 2.4. Handling of Mixed Populations

Several studies enrolled heterogeneous populations varying by age, metabolic status, periodontal condition, or disease severity. Where studies reported disaggregated salivary CRP values by relevant subgroups (e.g., MetS vs. controls, obese vs. normal weight, diabetic vs. non-diabetic), subgroup-specific data were extracted for analysis. This approach was applied to studies stratifying by metabolic syndrome components such as obesity [43,44,45,49,50,51,52,53,54,55,58,59,60], T2D [43,46,48,52,54,55], CVD [44,47,56,57,58], or hypertension [60].

When studies involved mixed cardiometabolic populations but only reported combined CRP values [43,44,45,49,50,51,52,53,54,55], the aggregated data were included with this limitation documented. For longitudinal studies with repeated measures [43,47,48,55,57,59,61], the time point most relevant to cardiometabolic assessment was selected.

When studies reported both aggregated cardiometabolic group data and stratified subgroup data, an extraction hierarchy was applied. Subgroup-specific data were preferentially extracted whenever available (e.g., obese vs. non-obese, T2D vs. controls, CVD vs. controls), as these provide greater phenotypic specificity and reduce clinical heterogeneity. Aggregated values were only used when subgroup-level data were not reported or could not be derived from the published results.

For studies reporting multiple metabolic phenotypes within the same cohort, each phenotype was entered separately into the corresponding subgroup meta-analysis, while a single representative comparison was used for the overall pooled analysis to avoid double-counting of participants.

Where participants had concurrent inflammatory conditions such as periodontitis [56,57,58], we prioritized the extraction of metabolically relevant groups and noted periodontal inflammation as a potential confounder in the quality assessment. The substantial heterogeneity observed in the pooled analysis (I^2^ = 98.91%) reflects, in part, the population diversity across studies (Figure 1).

### 2.5. Saliva Collection and CRP Assay Methods

The main methods of saliva collection included unstimulated whole saliva [43,44,45,46,47,48,49,50,51,52,53,54,55,56,57,59,61] and saliva obtained under standardized conditions (fasting state, morning collection) [43,45,46,49,50,53,54,55,58], with most studies processing samples through centrifugation before storage at −20 °C or −80 °C.

### 2.6. Statistical Analysis

All meta-analyses were conducted using a random-effects model due to expected clinical and methodological heterogeneity across studies. Standardized mean differences (SMDs) were computed to account for varying measurement scales. Where CRP levels were reported as medians with interquartile ranges, mean and standard deviation values were estimated using validated statistical conversions.

Between-study heterogeneity was assessed using Cochran’s Q test, the I^2^ statistic (interpreted as low, moderate, and high at approximately 25%, 50%, and 75%, respectively), and Tau^2^ to estimate between-study variance. Statistical significance was defined as *p* < 0.05. Publication bias was evaluated via funnel plot asymmetry and tested with Egger’s and Begg’s tests.

### 2.7. Subgroup Analysis

Pre-specified subgroup analyses were conducted to explore potential sources of heterogeneity and to examine whether salivary CRP levels differed according to specific metabolic phenotypes and assay methodology. As none of the included studies assessed metabolic syndrome as a unified clinical entity using standardized composite definitions, subgroup analyses were performed based on individual metabolic components, including obesity, T2D, and CVD. However, meta-analysis for the hypertension subgroup was not feasible because only a single study investigated hypertension as an outcome [60].

Additional subgroup analyses were conducted for studies reporting multiple metabolic or inflammatory subgroups (obesity, T2D, heart disease and hypertension); subgroup-specific effect estimates were extracted where available; otherwise, aggregated data were used.

Heterogeneity within each subgroup was quantified using the I^2^ statistic, and differences in pooled effect estimates across subgroups were examined descriptively to assess whether clinical phenotype or assay methodology contributed to variability in effect size.

### 2.8. Sensitivity Analysis

A leave-one-out sensitivity analysis was conducted to evaluate the robustness of the overall pooled estimate and to identify the influence of individual studies on the summary effect size and heterogeneity. In this analysis, each study was systematically removed from the meta-analysis one at a time, and the pooled standardized mean difference (SMD), 95% confidence interval (CI), and I^2^ statistic were recalculated for the remaining studies. This procedure allowed for the assessment of whether the overall conclusion was disproportionately driven by any single study. The stability of the pooled estimate was determined by observing the range of SMDs and the consistency of the direction and statistical significance of the effect after each exclusion.

## 3. Results

### 3.1. Study Selection and Characteristics

A total of 863 records were identified through database searching. Following the removal of 392 duplicates, 471 records underwent title and abstract screening. At this stage, review articles (*n* = 39), meta-analyses (*n* = 16), case reports (*n* = 30), case series (*n* = 26), and abstracts without available full texts (*n* = 5) were excluded. Eighty-five studies were subsequently assessed for full-text eligibility. Of these, 66 records were excluded after full-text review due to irrelevance to the research question (*n* = 42), insufficient data (*n* = 16), or animal and in vitro study design (*n* = 8). Ultimately, 19 studies met the inclusion criteria and were included in the quantitative synthesis, comprising a total of 3265 participants (Figure 1) [33,43,45,46,47,48,49,50,51,52,53,54,55,56,57,58,59,60,61].

The included studies represented diverse populations across five continents and 15 countries. Studies were conducted in Asia [43,49], Africa [44,45,48], Middle East [49,55], Europe [47,52,54,59,60], and North America [53,57]. Study populations included children and adolescents [43,45,49,50,53,54], adults [33,46,47,48,51,55,56,57,58,61], pregnant women [55], and elderly individuals [59]. Sample sizes ranged from 23 participants [57,61] to 600 participants [48], with several large cohorts derived from school-based screening programs [43,45,49,50,53,54]. Most studies em-ployed cross-sectional designs [45,46,47,50,53,54,58,59], with the remainder using case–control [33,51,52,56,57] or cohort designs [43,48,49,55,61] (Table 2).

The included studies examined individual cardiometabolic phenotypes related to metabolic syndrome, including obesity [43,44,45,49,50,51,52,53,54,55], T2D [43,46,48,53,61], CVD [33,47,58,59], hypertension [57], or combinations of these conditions (Table 2).

Salivary CRP concentrations were quantified using validated immunoassay techniques, predominantly high-sensitivity enzyme-linked immunosorbent assays (ELISA) [43,44,45,46,47,49,53,55,57,58,59] and multiplex bead-based platforms [33,50,52,54,61]. Methodological quality assessed using the Newcastle–Ottawa Scale ranged from moderate to high (scores 4–9), indicating a generally acceptable methodology across included studies [40,41,42] (Table 2).

### 3.2. Overall Meta-Analysis of Salivary CRP

Nineteen studies involving 3265 participants (1399 individuals with metabolic conditions and 1857 healthy controls) compared salivary CRP concentrations between groups [33,43,45,46,47,48,49,50,51,52,53,54,55,56,57,58,59,60,61] (Table 3).

**Figure 2:**
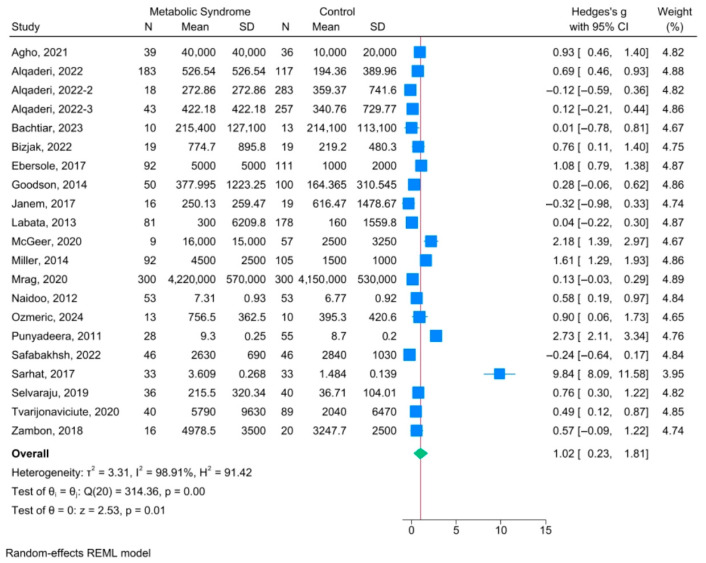
Forest plot displaying the standardized mean difference (Hedges’ g) of salivary CRP levels between individuals with metabolic syndrome and healthy controls [33,43,44,45,46,47,48,49,50,51,52,53,54,55,56,57,58,59,61].

Absolute salivary CRP concentrations demonstrated substantial variability across studies, ranging from 9.3 pg/mL to >420,000 pg/mL in metabolic disease groups and from 7.3 pg/mL to 415,000 pg/mL in control groups, representing a >40,000-fold difference [43,44,45,49,50,51,52,53,54,55,58,59,61]. The observed heterogeneity (I^2^ = 98.91%; *p* < 0.001) likely reflects differences in CRP assay methodologies, reporting units, saliva collection protocols, and the metabolic phenotypes represented across study populations. Random-effects meta-analysis demonstrated statistically significant higher salivary CRP levels in individuals with any component of metabolic syndrome compared with healthy controls (standardized mean difference [SMD] = 1.02; 95% confidence interval [CI]: 0.23–1.81; *p* = 0.01) (Figure 2).

Most studies reported positive associations between metabolic conditions and elevated salivary CRP. Several studies demonstrated large effect sizes, including McGeer et al. [59] (g = 2.18), Punyadeera et al. [58] (g = 2.73), and Miller et al. [56] (g = 1.61). In contrast, a minority of studies reported non-significant or negative associations, including Janem et al. [43], Alqaderi et al. [49], and Safabakhsh et al. [51]. Sarhat et al. [44] was identified as a prominent outlier, reporting an exceptionally large effect size (g = 9.84), contributing substantially to overall heterogeneity.

### 3.3. Subgroup Analysis: Obesity

**Figure 3:**
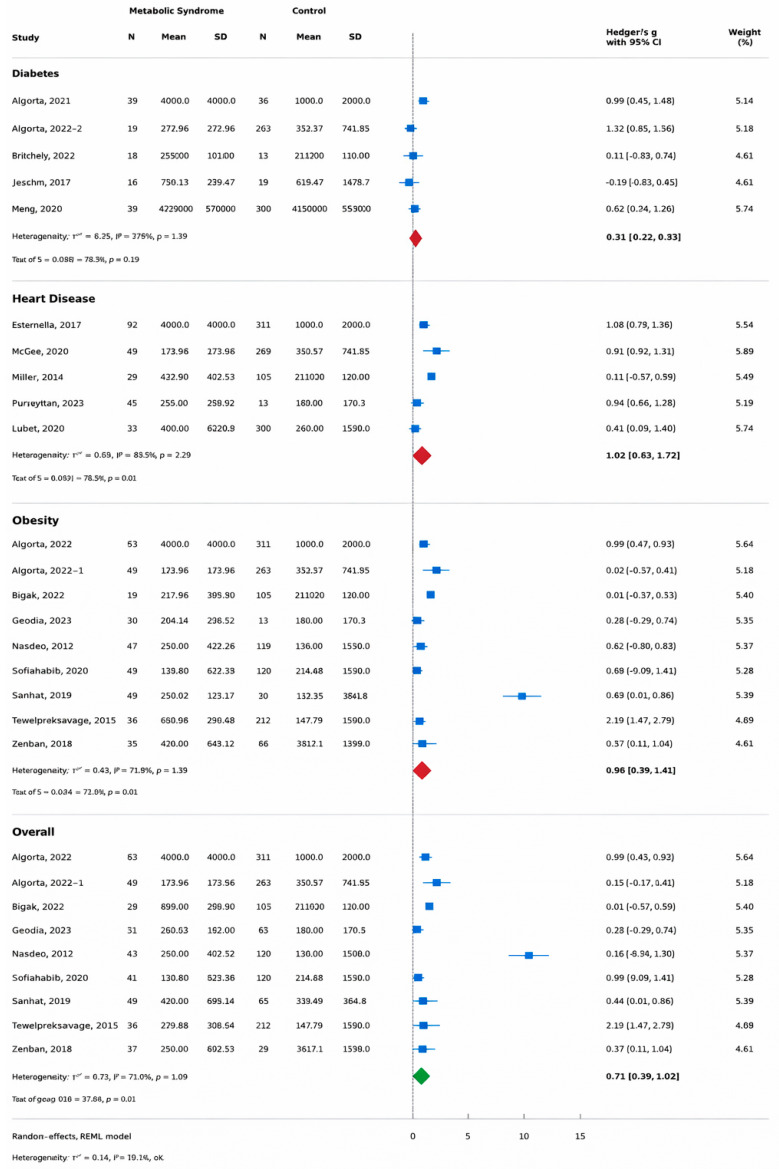
Overall and subgroup random-effects (REML) meta-analysis of salivary C-reactive protein (CRP). Forest plot comparing salivary CRP levels between individuals with metabolic conditions and healthy controls, stratified by metabolic phenotype (diabetes, heart disease, obesity). Effect sizes are reported as Hedges’ g (standardized mean differences) with 95% confidence intervals. The overall pooled estimate across all included comparisons showed higher salivary CRP in metabolic conditions (g = 0.71; 95% CI: 0.39–1.02; z = 4.40; *p* < 0.001), with substantial heterogeneity (τ^2^ = 0.44; I^2^ = 92.70%). Subgroup analyses showed no significant difference for diabetes (5 studies; *n* = 1034; g = 0.16; 95% CI: −0.22 to 0.53; z = 0.82; *p* = 0.41; I^2^ = 70.50%), but significant associations for heart disease (5 studies; *n* = 808; g = 1.02; 95% CI: 0.31–1.72; z = 2.81; *p* = 0.005; I^2^ = 94.68%) and obesity (10 studies; *n* = 1293; g = 0.90; 95% CI: 0.39–1.41; z = 3.46; *p* < 0.001; I^2^ = 93.27%). A significant difference between subgroups was observed (Qb(2) = 37.88; *p* < 0.001) [33,43,45,61].

Ten studies comprising 1293 participants (526 overweight or obese individuals and 693 controls) evaluated salivary CRP concentrations in relation to obesity status (Figure 3) [43,44,45,49,50,51,52,53,54,55]. The pooled random-effects meta-analysis demonstrated significantly higher salivary CRP levels in individuals with obesity compared with normal-weight controls (g = 0.90; 95% CI: 0.39–1.41; z = 3.46; *p* < 0.001).

Between-study heterogeneity was substantial (τ^2^ = 0.59; I^2^ = 93.27%), indicating considerable variability across studies in the magnitude of the effect.

While nine of the eleven studies reported positive associations between obesity and salivary CRP, only one study found a negative association [43]. Zambon et al. [55], which examined salivary CRP in pregnancies complicated by obesity and gestational diabetes, was included in the obesity subgroup analysis accordingly.

### 3.4. Subgroup Analysis: Cardiovascular Disease

Five studies involving 808 participants (371 individuals with CVD and 437 controls) assessed salivary CRP concentrations in relation to cardiovascular disease status [33,47,58,59] (Figure 3). The pooled random-effects analysis demonstrated significantly higher salivary CRP levels in individuals with cardiovascular disease (g = 1.02; 95% CI: 0.31–1.72; z = 2.81; *p* = 0.005). Heterogeneity within this subgroup was substantial (τ^2^ = 0.60; I^2^ = 94.68%), indicating marked between-study variability.

### 3.5. Subgroup Analysis: Type 2 Diabetes

Five studies comprising 1034 participants compared salivary CRP concentrations between individuals with T2D (*n* = 383) and healthy controls (*n* = 651) [43,46,48,53,61] (Figure 3). The pooled random-effects analysis did not demonstrate a statistically significant difference in salivary CRP levels between groups (g = 0.16; 95% CI: −0.22 to 0.53; z = 0.82; *p* = 0.41). Moderate heterogeneity was observed within this subgroup (τ^2^ = 0.12; I^2^ = 70.50%), suggesting more consistency across studies compared with the obesity and cardiovascular disease subgroups.

Mrag et al. [48], which contributed the largest sample size within this subgroup, reported no statistically significant difference in salivary CRP levels between individuals with T2D and controls. Similarly, Janem et al. [43] reported no significant difference in salivary CRP between obese individuals with T2D and control participants.

### 3.6. Sensitivity Analysis

A series of sensitivity analyses were conducted to evaluate the robustness of the pooled effect estimate and explore potential sources of heterogeneity.

In the leave-one-out analysis, the pooled standardized mean difference (SMD) ranged from 0.52 (95% CI: 0.28 to 0.76; excluding Sarhat 2017 [44]) to 0.77 (95% CI: 0.45 to 1.08; excluding Selvaraju 2019 [50]). In all iterations, the direction of effect remained positive and statistically significant (*p* < 0.001), indicating that no single study reversed the overall association (Table 4).

Given the presence of studies reporting extreme salivary CRP concentrations, additional sensitivity analyses were performed. Exclusion of the most extreme statistical outlier (Sarhat 2017 [44]; Hedges’ g = 9.84) reduced the pooled SMD to 0.52 (95% CI: 0.28 to 0.76), with heterogeneity decreasing from I^2^ = 92.3% to I^2^ = 87.4%. When all four studies reporting extreme absolute CRP values (Sarhat 2017 [44], Mrag 2020 [48], Bachtiar 2023 [61], and Agho 2021 [46]) were excluded, the pooled SMD was 0.55 (95% CI: 0.28 to 0.83) with I^2^ = 87.3% (Table 5). In both scenarios, the association remained positive and statistically significant, although attenuated in magnitude.

To assess whether studies involving periodontal disease influenced the pooled estimate, a sensitivity analysis excluding Ebersole 2017 [56], Ozmeric 2024 [57], and Punyadeera 2011 [58] was conducted. The resulting pooled SMD was 0.71 (95% CI: 0.37 to 1.05) with I^2^ = 93.0%, which was comparable to the primary analysis (Table 4), suggesting that inclusion of periodontal cohorts did not substantially alter the overall findings.

To further contextualize heterogeneity, 95% prediction intervals were calculated. The overall prediction interval ranged from −0.71 to 2.14, indicating that although the average pooled effect was positive, the true effect in a future study could plausibly range from a small negative to a large positive value. Subgroup prediction intervals were similarly wide: Diabetes (−1.10 to 1.41), Heart Disease (−1.70 to 3.73), and Obesity (−0.97 to 2.77) (Table 6).

Exploratory meta-regression analyses were undertaken to investigate potential contributors to heterogeneity. Total sample size was not a statistically significant predictor of effect size (coefficient = −0.0017, *p* = 0.118). When cardiometabolic condition type was included as a categorical covariate, borderline differences were observed between Heart Disease and Diabetes (*p* = 0.054) and between Obesity and Diabetes (*p* = 0.077), suggesting that the underlying disease phenotype may partially account for between-study variability (Table 7).

### 3.7. Publication Bias

**Figure 4:**
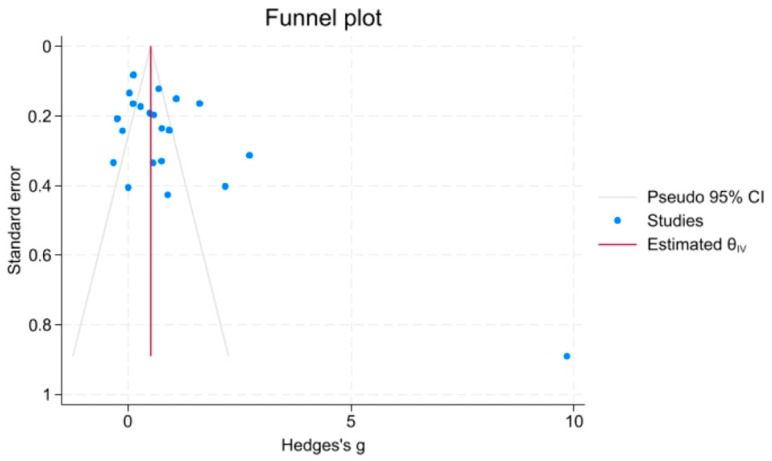
Funnel plot for publication bias assessment of salivary CRP in MetS vs. healthy controls. Each dot represents an included study plotted by effect size (Hedges’ g) against standard error. The vertical line represents the estimated overall effect (${\hat{\theta }}_{\mathrm{iv}}$), and the pseudo 95% confidence interval bounds are shown in gray. The funnel plot demonstrates visible asymmetry with clustering of studies on the left side and a notable outlier on the right (Hedges’ g ≈ 10). While Egger’s regression test (*p* = 0.421) and Begg’s rank correlation test (*p* = 0.553) did not reach statistical significance, the visual asymmetry and limited statistical power with only 19 studies suggest that publication bias cannot be confidently ruled out.

Visual inspection of the funnel plot suggested some asymmetry, with clustering of smaller studies and the presence of an extreme outlier (Figure 4). However, formal statistical testing did not indicate significant publication bias (Egger’s regression test: *p* = 0.421; Begg’s rank correlation test: *p* = 0.553). Given the small number of included studies (*n* = 19) and the presence of substantial heterogeneity, the power of these tests is limited, and publication bias cannot be completely excluded.

## 4. Discussion

This systematic review and meta-analysis demonstrate that salivary C-reactive protein (CRP) levels are elevated across a range of metabolic disorders, including obesity, type 2 diabetes mellitus (T2D), hypertension, and cardiovascular disease (CVD), compared with healthy controls. The overall pooled effect size indicated a notable increase in salivary CRP among individuals with metabolic dysfunction, although this finding was accompanied by high between-study heterogeneity (I^2^ = 98.91%). Subgroup analyses demonstrated phenotype-specific differences in the association between salivary CRP and metabolic disorders. Significant pooled elevations in salivary CRP were observed for both obesity and cardiovascular disease, whereas no statistically significant association was identified for T2D. Despite these significant findings, heterogeneity remained substantial within the obesity and CVD subgroups, indicating wide variability in effect size magnitude across studies [33,43,45,46,47,48,49,50,51,52,53,54,55,56,57,58,59,60,61]. In contrast, the T2D subgroup exhibited comparatively lower heterogeneity, suggesting greater consistency but a weaker overall association, which is consistent with recent reviews of salivary biomarkers [61,62,63].

This variability likely reflects differences in salivary CRP measurement methodologies, reporting units, and saliva collection protocols, with absolute concentrations of >40,000-fold difference between the MetS groups versus control groups [33,43,45,46,47,48,49,50,51,52,53,54,55,56,57,58,59,60,61].

The observed elevation of salivary CRP across cardiometabolic conditions aligns with current understanding of MetS as a chronic low-grade inflammatory state [6,7,12]. Inflammatory pathways involving cytokines such as IL-6, tumor necrosis factor-α, and monocyte chemoattractant protein-1 drive hepatic CRP synthesis and contribute to endothelial dysfunction, insulin resistance, and atherosclerosis [12,13,14,18,19]. CRP is therefore not merely a passive biomarker but a downstream integrator of inflammatory activity central to metabolic dysregulation [15,16]. The detection of this inflammatory signal in saliva supports previous evidence demonstrating moderate-to-strong correlations between salivary and serum CRP concentrations across diverse clinical contexts [25,26,27,35,58].

The robustness of the pooled estimate was systematically evaluated through multiple sensitivity analyses. The leave-one-out analysis demonstrated that no single study was solely responsible for the observed association, with pooled SMDs ranging from 0.52 to 0.77 across all iterations. Notably, the exclusion of Sarhat et al. (2017) [44], which reported an exceptionally large effect size (Hedges’ g = 9.84), produced the greatest attenuation of the pooled estimate (from 0.71 to 0.52), although the association remained statistically significant. Similarly, excluding all four studies reporting extreme CRP concentrations did not eliminate the positive association (SMD = 0.55, 95% CI: 0.28 to 0.83), further supporting the consistency of the finding.

Subgroup analysis by metabolic phenotype provided partial insight into sources of heterogeneity, though with important limitations. T2D demonstrated the lowest heterogeneity among subgroups, suggesting that salivary CRP elevation may be more consistent in established hyperglycemic states, possibly reflecting more uniform inflammatory mechanisms associated with chronic glucose dysregulation [43,46,48,53,61] or more standardized diagnostic criteria for diabetes compared to other metabolic conditions [52,63]. In contrast, obesity and CVD exhibited substantial heterogeneity, which may reflect differences in adiposity distribution (visceral vs. subcutaneous), metabolic health status [43,44,45,49,50,51,52,53,54,55,58,59,60], disease severity, and the presence of comorbidities such as CVD across study populations [33,47,58,59]. However, the lack of statistically significant associations between salivary CRP and specific metabolic phenotypes in subgroup analyses, combined with persistent high heterogeneity even within disease categories, indicates that phenotype-specific stratification alone cannot fully explain the observed variability. This reinforces the likelihood that methodological inconsistencies in saliva collection, assay techniques, and inadequate control for confounders such as periodontal disease contribute significantly to effect size dispersion across the evidence base and limit our ability to draw condition-specific conclusions about salivary CRP as a biomarker.

The sensitivity analysis excluding studies with a known periodontal disease [56,57,58] component yielded results comparable to the primary analysis (SMD = 0.71, 95% CI: 0.37 to 1.05), indicating that oral inflammatory cohorts did not disproportionately drive the pooled effect. Nevertheless, residual confounding by periodontal status remains plausible, as most included studies did not comprehensively assess or adjust for oral health conditions.

The 95% prediction interval for the overall effect (−0.71 to 2.14) warrants cautious interpretation. Although the confidence interval indicates a statistically significant average association, the prediction interval crossing zero suggests that the true effect in a future study could plausibly be null. This finding is consistent with the substantial between-study heterogeneity and underscores that salivary CRP elevation may not be uniformly observed across all populations or clinical contexts.

Exploratory meta-regression analyses did not identify total sample size as a significant contributor to heterogeneity. Condition type demonstrated borderline associations, with heart disease and obesity tending toward larger effect sizes compared with diabetes. However, given the limited number of studies within subgroups and the exploratory nature of these analyses, these findings should be interpreted cautiously.

Several methodological and clinical factors likely contributed to this heterogeneity. Methodological variability was substantial, including differences in saliva collection protocols (stimulated versus unstimulated, fasting versus non-fasting), sample processing and storage conditions, and CRP assay platforms. Although both ELISA-based and multiplex bead-based assays demonstrated significant elevations in salivary CRP, heterogeneity remained high within each subgroup, suggesting that analytical variability alone does not fully explain the observed dispersion of effect sizes [43,44,46,47,48,51,55,58,59,60]. Differences in assay sensitivity, calibration ranges, and reporting units further complicate cross-study comparisons [24,25,26,27,28,29].

Clinical heterogeneity was also substantial. The included studies encompassed pediatric, adult, pregnant, and elderly populations, with wide variation in sex distribution, ethnicity, and comorbidity profiles [33,43,45,46,47,48,49,50,51,52,53,54,55,56,57,58,59,60,61]. The overall pooled analysis was therefore conceptualized to examine whether salivary CRP reflects a shared inflammatory signal across cardiometabolic disorders that comprise the MetS spectrum, rather than to represent a diagnosis-specific meta-analysis of formally defined MetS. Obesity, T2D, hypertension, and cardiovascular disease are linked through chronic low-grade systemic inflammation, insulin resistance, endothelial dysfunction, and cytokine-mediated hepatic CRP synthesis. CRP thus functions as an integrative downstream biomarker of cardiometabolic inflammatory burden rather than a disease-specific marker.

Importantly, none of the included studies applied a formal, unified diagnostic definition of metabolic syndrome based on established criteria such as NCEP ATP III, IDF, or WHO [5,11,21]. Instead, most investigations examined individual cardiometabolic components, including obesity, T2D, or cardiovascular disease. As MetS is defined by the co-occurrence of multiple metabolic abnormalities—typically central obesity, dyslipidemia, hypertension, and impaired glucose regulation—the absence of standardized diagnostic criteria indicates that pooled estimates reflect inflammatory burden across heterogeneous metabolic phenotypes rather than metabolic syndrome as a discrete clinical entity. Consequently, the specificity of inferences regarding salivary CRP as a biomarker for metabolic syndrome per se is limited [11,13]. The overall pooled estimate should therefore be interpreted as reflecting inflammatory burden across cardiometabolic disorders rather than as a proxy for formally diagnosed metabolic syndrome.

A further critical limitation is inadequate control for confounding factors, particularly oral inflammatory conditions. Periodontal disease is a well-established source of elevated salivary CRP through local immune activation and gingival crevicular fluid transudation, independent of systemic inflammation [37,58]. Given the strong bidirectional relationship between periodontal disease and metabolic disorders such as obesity and diabetes, elevated salivary CRP observed in metabolic populations may partly reflect undiagnosed or unadjusted oral inflammation rather than systemic metabolic dysfunction [12,37,56,57,58]. Most included studies did not perform comprehensive periodontal assessments or adjust for oral health status, representing a plausible alternative explanation for both elevated CRP levels and inter-study heterogeneity.

Additional confounders, including smoking, age, sex, medication use, acute infections, and socioeconomic factors, were inconsistently reported and rarely adjusted for across studies [17,63]. These variables are known to influence systemic inflammatory markers and may have contributed to variability in salivary CRP concentrations. The lack of standardized confounder adjustment further limits causal interpretation and underscores the need for more rigorous study designs.

The distinction between analytical validity and clinical utility is particularly important when interpreting these findings. Analytical validity refers to the ability to reliably and accurately measure CRP in saliva, which is supported by multiple studies demonstrating correlations between salivary and serum CRP concentrations [25,26,27,35,54]. In contrast, clinical utility requires evidence that salivary CRP improves disease detection, risk stratification, or patient outcomes beyond existing clinical tools. Although our meta-analysis confirms that salivary CRP is elevated in metabolic conditions, the substantial overlap in salivary CRP values between cases and controls and the absence of standardized thresholds preclude its use as a standalone diagnostic or screening tool at present [30,36].

No included studies evaluated the prognostic value of salivary CRP for incident metabolic syndrome, cardiovascular events, or diabetes progression, nor did they assess whether salivary CRP-guided interventions improve clinical outcomes [8,9,11]. Without prospective validation and clearly defined clinical action thresholds, salivary CRP remains a promising research biomarker rather than a clinically actionable test. This distinction is critical to avoid premature translation into practice without sufficient evidence.

Our findings are broadly consistent with recent narrative and systematic reviews on salivary biomarkers, which have highlighted the potential of salivary CRP while emphasizing the need for methodological standardization and cautious interpretation [35,37,63]. However, this meta-analysis extends previous work by providing the first quantitative synthesis of salivary CRP across metabolic disease phenotypes and by explicitly characterizing the magnitude and sources of heterogeneity that limit clinical application. By demonstrating that heterogeneity is intrinsic to the current evidence base, rather than a minor methodological inconvenience, this study reframes heterogeneity as a central finding that must be addressed before clinical implementation can be considered.

Several priorities for future research emerge from this analysis. First, studies should explicitly recruit participants meeting standardized diagnostic criteria for metabolic syndrome, as defined by international consensus guidelines, to allow condition-specific evaluation of salivary CRP [5,39]. Second, harmonization of saliva collection protocols and CRP assay methodologies is essential, including standardized timing, fasting status, storage conditions, assay sensitivity, and reporting units [24,25,26,27,28,29]. Third, rigorous control for confounding factors, particularly periodontal disease, should be incorporated through comprehensive oral examinations or validated inflammatory markers [37,60]. Finally, prospective longitudinal studies are needed to establish temporal relationships, define clinically meaningful thresholds, and determine whether salivary CRP predicts disease onset or progression or responds to therapeutic interventions such as weight loss, glycemic control, or anti-inflammatory treatments [6,7,18,19].

## 5. Conclusions

In conclusion, this meta-analysis provides exploratory evidence that salivary CRP tends to be elevated across a range of metabolic disorders, suggesting a possible association with systemic inflammatory burden related to metabolic dysfunction. The findings highlight the promise of salivary CRP as an accessible, non-invasive candidate biomarker that warrants further investigation. However, the between-study heterogeneity and variability in study design and measurement methods emphasize the need to standardize salivary CRP assessment. The inclusion of studies examining related but distinct metabolic conditions limits the specificity of inferences regarding MetS itself. Future high-quality studies employing harmonized definitions of metabolic syndrome, standardized salivary CRP assays, and prospective designs are required to clarify consistency, establish clinically meaningful thresholds, evaluate predictive performance, and determine whether salivary CRP has a role in metabolic risk stratification and early prevention strategies.

## Figures and Tables

**Table 1:** Summary of Major Diagnostic Criteria with Examples.

Organizations/Definitions	Required Components	Diagnostic Thresholds (Examples)
NCEP ATP III (2001) [1]	Any 3 of: waist circumference, fasting glucose, BP, TG, HDL	Waist ≥ 102/88 cm (M/F), FPG ≥ 100 mg/dL
WHO (1998) [5]	Insulin resistance + 2 of: obesity, hypertension dyslipidemia	Waist/hip ratio, BP ≥ 140/90, TG ≥ 150 mg/dL, HDL < 35/39 mg/dL (M/F)
IDF (2005) [21]	Central obesity (mandatory) + 2 of: raised TG, low HDL, high BP, raised fasting glucose	Waist (ethnic-specific), TG ≥ 150 mg/dL

**Table 2:** Summary of Study Design, Population Characteristics and Quality Assessment of the Included Studies.

Study	Country	Design	Sample Size	Age (Years)	Gender (M/F%)	NOS
Miller, 2010 [33]	USA	Case–control	*n* = 197	42–66	42.9 vs. 57.1	8
Janem, 2017 [43]	USA	Cohort	*n* = 35	10–19	NA	4
Naidoo, 2012 [45]	South Africa	Cross sectional	*n* = 170	7–11	41.1 vs. 58.9	8
Agho, 2021 [46]	Nigeria	Cross-sectional	*n* = 75	20–70	57.4 vs. 42.6	7
Labat, 2013 [47]	France	Cross-sectional	*n* = 255	50–70	69 vs. 31	9
Mrag, 2020 [48]	Tunisia	Cohort	*n* = 600	50–70	NA	8
Alqaderi, 2022 [49]	Kuwait	Cohort	*n* = 353	9–11	61.3 vs. 38.7	8
Selvaraju, 2019 [50]	USA	Cross-sectional	*n* = 76	6–10	58 vs. 42	5
Safabakhsh, 2022 [51]	Iran	Case–control	*n* = 92	20–40	34.7 vs. 65.2	8
Bizjak, 2022 [52]	Germany	Case–control	*n* = 38	5–19	50 vs. 50	6
Goodson, 2014 [53]	USA and Kuwait	Cross-sectional	*n* = 150	9–12	34.4 vs. 65.6	6
Tvarijonaviciute, 2020 [54]	Spain	Cross-sectional	*n* = 129	9–12	53.4 vs. 46.6	6
Zambon, 2018 [55]	Italy	Cohort	*n* = 36	18–40	F:100	8
Ebersole, 2017 [56]	USA	Case–control	*n* = 203	40–57	70.7 vs. 29.3	7
Ozmeric, 2024 [57]	Cyprus	Case–control	*n* = 23	20–70	52.1 vs. 48	6
Punyadeera, 2011 [58]	Australia	Cross-sectional	*n* = 83	18–70	NA	6
McGeer, 2020 [59]	Canada	Cross-sectional	*n* = 66	16–92	42.3 vs. 57.7	5
Bachtiar, 2024 [61]	Indonesia	Cohort	*n* = 23	30–70	47 vs. 53	5

**Table 3:** A summary of the salivary collection methods, assay platforms and key findings of the included studies.

Study	Outcome Assessed	Saliva Collection Method	Assay Platform	Key Findings
Miller, 2010 [33]	Cardiovascular disease	Serum and unstimulated saliva	Luminex^®^ IS-100 instrument (Luminex^®^ Corp., Austin, TX, USA	Salivary CRP levels were elevated in patients with acute myocardial infarction.
Janem, 2017 [43]	Obesity and T2D	Unstimulated whole saliva	ELISA (Salimetrics, State College, PA, USA)	Salivary CRP levels were elevated in individuals with obesity, particularly when GDM was present.
Sarhat, 2017 [44]	Obesity	Unstimulated whole saliva	ELISA (Salimetrics, State College, PA, USA)	Salivary CRP levels were elevated in individuals with obesity.
Naidoo, 2012 [45]	Obesity	Unstimulated whole saliva	ELISA (Salimetrics, State College, PA, USA)	Salivary CRP levels were elevated in individuals with obesity.
Agho, 2021 [46]	T2D	Unstimulated whole saliva collected using spit method every six seconds for 5 min	ELISA test kit (Monobind Inc. Lake Forest, CA 52,630 USA)	Salivary CRP levels were elevated in individuals with T2D and showed a positive correlation with serum HbA1c.
Labat, 2013 [47]	Cardiovascular disease	Unstimulated whole saliva	ELISA Salimetrics (Suffolk, UK).	Salivary CRP levels were elevated in individuals with cardiovascular disease, supporting their potential role in cardiovascular risk assessment.
Mrag, 2020 [48]	T2D	Unstimulated whole saliva	Multiplex assay (MILLIPLEX MAP Human)	Salivary CRP levels were not elevated in individuals with T2D.
Alqaderi, 2022 [49]	Obesity and intermediate hyperglycemia	Unstimulated whole saliva and stimulated saliva	ELISA (Peprotech, NJ, USA)	Salivary CRP levels were elevated in individuals with increased adiposity and, together with salivary insulin, showed strong predictive value for metabolic risk.
Selvaraju, 2019 [50]	Obesity	Unstimulated whole saliva	Luminex IS-100 instrument assay, (catalog # LOBM000, R&D systems, Boston, MA, USA)	Salivary CRP levels were elevated in individuals with obesity and demonstrated superior discriminatory performance compared with other biomarkers.
Safabakhsh, 2022 [51]	Obesity	Unstimulated whole saliva	ELISA (Human Interleukin ELISA Kit, Hangzhou Eastbiopharm, Co., Hangzhou, China)	Salivary CRP levels were not elevated in individuals with obesity.
Bizjak, 2022 [52]	Obesity	Unstimulated whole saliva	Luminex 200™ system (Luminex, Austin, TX, USA)	Salivary CRP levels were elevated in children with obesity.
Goodson, 2014 [53]	Obesity and T2D	Unstimulated whole saliva	ELISA (Salimetrics, State College, PA, USA)	Salivary CRP levels were elevated in children with obesity and in children with T2D.
Tvarijonaviciute, 2020 [54]	Obesity	Unstimulated whole saliva	Luminex 200 platform (Luminex, Austin, TX, USA)	Salivary CRP levels were elevated with increasing BMI.
Zambon, 2018 [55]	Obesity	Unstimulated whole saliva	ELISA (Biosource, Invitrogen Corporation, Carlsbad, CA, USA)	Salivary CRP levels were elevated in relation to maternal BMI and gestational diabetes.
Ozmeric, 2024 [57]	Hypertension	Unstimulated whole saliva	ELISA (Elabscience Biotechnology Inc., Wuhan, China)	Salivary CRP levels were elevated in individuals with hypertension.
Punyadeera, 2011 [58]	Cardiovascular disease	Unstimulated whole saliva and stimulated saliva	ELISA (AL233C, Perkin Elmer^®^, Waltham, MA, USA)	Salivary CRP levels were elevated in patients with cardiac disease.
McGeer, 2020 [59]	Cardiovascular disease	Unstimulated whole saliva	ELISA	Salivary CRP levels were elevated in patients with cardiac disease.
Bachtiar, 2024 [61]	T2D	Unstimulated whole saliva	Luminex 200™ system (Luminex, Austin, TX, USA)	Salivary CRP levels were elevated in individuals with diabetes.

ELISA = Enzyme-Linked Immunosorbent Assay, AMI = acute myocardial infarction, T2D = Type 2 diabetes; GDM = gestational diabetes.

**Table 4:** Leave-One-Out Sensitivity Analysis.

Study Excluded	SMD	95% CI	I^2^ (%)	*p*-Value
Miller, 2010 [33]	1.25	[0.73, 1.77]	95.6	<0.001
Sarhat, 2017 [44]	0.97	[0.50, 1.43]	95.1	<0.001
Naidoo, 2012 [45]	1.41	[0.83, 1.99]	96.5	<0.001
Agho, 2021 [46]	1.38	[0.81, 1.95]	96.5	<0.001
Labat, 2013 [47]	1.46	[0.87, 2.05]	96.3	<0.001
Mrag, 2020 [48]	1.46	[0.90, 2.02]	96.4	<0.001
Alqaderi, 2022 [49]	1.39	[0.83, 1.95]	96.5	<0.001
Selvaraju, 2019 [50]	1.39	[0.82, 1.96]	96.5	<0.001
Safabakhsh, 2022 [51]	1.47	[0.90, 2.03]	96.3	<0.001
Bizjak, 2022 [52]	1.41	[0.83, 1.99]	96.5	<0.001
Goodson, 2014 [53]	1.47	[0.86, 2.07]	95.8	<0.001
Tvarijonaviciute, 2020 [54]	1.37	[0.82, 1.93]	96.5	<0.001

Overall pooled SMD = 1.34 (95% CI: 0.81 to 1.88), I^2^ = 96.23%.

**Table 5:** Summary of Sensitivity Analyses.

Analysis	k	SMD	95% CI	95% PI	I^2^ (%)	τ^2^	*p*-Value
Overall (all 19 studies)	19	0.71	[0.41, 1.02]	[−0.71, 2.14]	92.3	0.44	<0.001
Excluding Sarhat 2017 [44] (outlier)	18	0.52	[0.28, 0.76]	[−0.54, 1.58]	87.4	0.24	<0.001
Excluding all extreme CRP studies	15	0.55	[0.28, 0.83]	[−0.61, 1.71]	87.3	0.26	<0.001
Excluding periodontal studies	15	0.71	[0.37, 1.05]	[−0.79, 2.21]	93.0	0.47	<0.001

Extreme CRP studies excluded: Sarhat 2017 [44], Agho 2021 [46], Mrag 2020 [48], Bachtiar 2023 [61]. Periodontal studies excluded: Ebersole 2017 [56], Ozmeric 2024 [57], Punyadeera 2011 [58].

**Table 6:** Prediction Intervals by Subgroup.

Subgroup	k	SMD	95% CI	95% PI	I^2^ (%)	τ^2^
Diabetes	5	0.16	[−0.22, 0.53]	[−1.10, 1.41]	70.5	0.12
Heart Disease	5	1.02	[0.31, 1.72]	[−1.70, 3.73]	94.7	0.60
Obesity	10	0.90	[0.39, 1.41]	[−0.97, 2.77]	93.3	0.59
Overall	21	0.71	[0.41, 1.02]	[−0.71, 2.14]	92.3	0.44

PI = prediction interval. The prediction interval estimates the range within which the true effect of a future study is expected to fall with 95% probability.

**Table 7:** Meta-Regression Results.

Covariate	Coefficient	SE	z	*p*-Value
**Model 1: Sample Size**				
Intercept	0.997	0.239	4.17	<0.001
Total sample size	−0.0017	0.0011	−1.56	0.118
**Model 2: Condition Type**				
Intercept (Diabetes)	0.139	0.322	0.43	0.667
Heart Disease vs. Diabetes	0.867	0.449	1.93	0.054
Obesity vs. Diabetes	0.698	0.395	1.77	0.077
Hypertension vs. Diabetes	0.758	0.859	0.88	0.378

Meta-regression using random-effects model with restricted maximum likelihood (REML) estimation. Model 1 tested sample size as a continuous covariate. Model 2 tested condition type as a categorical covariate (reference: Diabetes).

## Data Availability

The original contributions presented in this study are included in the article/Supplementary Materials. Further inquiries can be directed to the corresponding author.

## Supplementary Materials

The following supporting information can be downloaded at: https://www.mdpi.com/article/10.3390/life16030403/s1. Supplementary File S1. Comprehensive Search Strategy Applied Across All Databases. Supplementary File S2. Excluded Articles with Reasons for Exclusion [1,3,10,16,18,21,25,26,29,30,31,32,33,35,37,64,65,66,67,68,69,70,71,72,73,74,75,76,77,78,79,80,81,82,83,84,85,86,87,88,89,90,91,92,93,94,95,96,97,98,99,100,101,102,103,104,105,106,107,108,109,110,111,112].

## Abbreviations

The following abbreviations are used in this manuscript:

MetS	Metabolic Syndrome
CRP	C-Reactive Protein
T2D	Type 2 diabetes
CVD	Cardiovascular disease
ELISA	enzyme-linked immunosorbent assay
NOS	Newcastle–Ottawa Scale
BMI	Body Mass Index
SMDs	Standardized mean differences

## References

[1] Samson S.L., Garber A.J. (2014). Metabolic syndrome. Endocrinol. Metab. Clin..

[2] Huang P. (2009). A comprehensive definition for metabolic syndrome. Dis. Model. Mech..

[3] Saklayen M.G. (2018). The Global Epidemic of the Metabolic Syndrome. Curr. Hypertens. Rep..

[4] Pigeot I., Ahrens W. (2025). Epidemiology of metabolic syndrome. Pflügers Archiv..

[5] Alberti K.G.M., Zimmet P., Shaw J. (2005). The metabolic syndrome—A new worldwide definition. Lancet.

[6] Hotamisligil G.S. (2006). Inflammation and metabolic disorders. Nature.

[7] Grundy S.M. (2008). Metabolic syndrome pandemic. Arterioscler. Thromb. Vasc. Biol..

[8] Wilson P.W., D’Agostino R.B., Parise H., Sullivan L., Meigs J.B. (2005). Metabolic syndrome as a precursor of cardiovascular disease and type 2 diabetes mellitus. Circulation.

[9] Mottillo S., Filion K.B., Genest J., Joseph L., Pilote L., Poirier P., Rinfret S., Schiffrin E.L., Eisenberg M.J. (2010). The metabolic syndrome and cardiovascular risk: A systematic review and meta-analysis. J. Am. Coll. Cardiol..

[10] Cameron A.J., Shaw J.E., Zimmet P.Z. (2004). The metabolic syndrome: Prevalence in worldwide populations. Endocrinol. Metab. Clin..

[11] Gesteiro E., Megía A., Guadalupe-Grau A., Fernandez-Veledo S., Vendrell J., González-Gross M. (2021). Early identification of metabolic syndrome risk: A review of reviews and proposal for defining pre-metabolic syndrome status. Nutr. Metab. Cardiovasc. Dis..

[12] Gregor M.F., Hotamisligil G.S. (2011). Inflammatory mechanisms in obesity. Annu. Rev. Immunol..

[13] Srikanthan K., Feyh A., Visweshwar H., Shapiro J.I., Sodhi K. (2016). Systematic review of metabolic syndrome biomarkers: A panel for early detection, management, and risk stratification in the west virginian population. Int. J. Med. Sci..

[14] Kim S.-H., Lee J.-W., Im J.-A., Hwang H.-J. (2011). Monocyte chemoattractant protein-1 is related to metabolic syndrome and homocysteine in subjects without clinically significant atherosclerotic cardiovascular disease. Scand. J. Clin. Lab. Investig..

[15] Pepys M.B., Hirschfield G.M. (2003). C-reactive protein: A critical update. J. Clin. Investig..

[16] Ansar W., Ghosh S. (2013). C-reactive protein and the biology of disease. Immunol. Res..

[17] Pearson T.A., Mensah G.A., Alexander R.W., Anderson J.L., Cannon R.O., Criqui M., Fadl Y.Y., Fortmann S.P., Hong Y., Myers G.L. (2003). Markers of inflammation and cardiovascular disease: Application to clinical and public health practice: A statement from the CDC and the AHA. Circulation.

[18] Ridker P.M., Buring J.E., Cook N.R., Rifai N. (2003). C-reactive protein, the metabolic syndrome, and risk of incident cardiovascular events: An 8-year follow-up of 14,719 initially healthy American women. Circulation.

[19] Festa A., D’Agostino R., Howard G., Mykkänen L., Tracy R.P., Haffner S.M. (2000). Chronic subclinical inflammation as part of the insulin resistance syndrome: The IRAS. Circulation.

[20] Sakkinen P.A., Macy E.M., Callas P.W., Cornell E.S., Hayes T.E., Kuller L.H., Tracy R.P. (1999). Analytical and biologic variability in measures of hemostasis, fibrinolysis, and inflammation. Am. J. Epidemiol..

[21] Pradhan A.D., Manson J.E., Rifai N., Buring J.E., Ridker P.M. (2001). C-reactive protein, interleukin 6, and risk of developing type 2 diabetes mellitus. JAMA.

[22] Ridker P.M., Rifai N., Clearfield M., Downs J.R., Weis S.E., Miles J.S., Gotto A.M. (2001). Measurement of C-reactive protein for targeting statin therapy in primary prevention. N. Engl. J. Med..

[23] Verma S., Li S.H., Badiwala M.V., Weisel R.D., Fedak P.W.M., Li R.K., Dhillon B., Mickle D.A.G. (2002). Endothelin antagonism and interleukin-6 inhibition attenuate proatherogenic effects of CRP. Circulation.

[24] Kilpatrick E.L., Bunk D.M. (2009). Reference measurement procedure development for CRP in human serum. Anal. Chem..

[25] Ouellet-Morin I., Danese A., Williams B., Arseneault L. (2011). Validation of a high-sensitivity assay for CRP in human saliva. Brain Behav. Immun..

[26] Out D., Hall R.J., Granger D.A., Page G.G., Woods S.J. (2012). Assessing salivary CRP: Longitudinal associations with systemic inflammation and CVD risk. Brain Behav. Immun..

[27] Dongiovanni P., Meroni M., Casati S., Goldoni R., Thomaz D.V., Kehr N.S., Galimberti D., Del Fabbro M., Tartaglia G.M. (2023). Salivary biomarkers: Novel noninvasive tools to diagnose chronic inflammation. Int. J. Oral. Sci..

[28] Goin Y., Fanous E., Pasternak Y., Prokocimer Z., Zagoory-Sharon O., Feldman R., Codick G., Waisbourd-Zinman O., Fried S., Livni G. (2021). Salivary CRP—A possible predictor of serum levels in pediatric acute respiratory illness. Eur. J. Pediatr..

[29] Malamud D., Rodriguez-Chavez I.R. (2011). Saliva as a diagnostic fluid. Dent. Clin. N. Am..

[30] Pfaffe T., Cooper-White J., Beyerlein P., Kostner K., Punyadeera C. (2011). Diagnostic potential of saliva: Current state and future applications. Clin. Chem..

[31] Javaid M.A., Ahmed A.S., Durand R., Tran S.D. (2016). Saliva as a diagnostic tool for oral and systemic diseases. J. Oral. Biol. Craniofac Res..

[32] Malon R.S.P., Sadir S., Balakrishnan M., Córcoles E.P. (2014). Saliva-based biosensors: Noninvasive monitoring tool for clinical diagnostics. BioMed Res. Int..

[33] Miller C.S., Foley J.D., Bailey A.L., Campell C.L., Humphries R.L., Christodoulides N., Floriano P.N., Simmons G., Bhagwandin B., Jacobson J.W. (2010). Current developments in salivary diagnostics. Biomark. Med..

[34] Cuevas-Córdoba B., Santiago-García J. (2014). Saliva: A fluid of study for OMICS. OMICS.

[35] Castagnola M., Picciotti P.M., Messana I., Fanali C., Fiorita A., Cabras T., Calò L., Pisano E., Passali G.C., Iavarone F. (2011). Potential applications of human saliva as diagnostic fluid. Acta Otorhinolaryngol..

[36] Bedi G.N., Acharya S., Kumar S., Mapari S.A. (2024). Salivary High-Sensitivity CRP and clinical relevance in modern medicine: Review. Cureus.

[37] Dezayee Z.M., Al-Nimer M.S. (2016). Saliva CRP as a biomarker of metabolic syndrome in diabetic patients. Indian. J. Dent. Res..

[38] Babaei M., Rezaei S., Saghafi Khadem S., Shirinbak I., Basir Shabestari S. (2022). Role of salivary CRP in systemic and oral disorders: A systematic review. Med. J. Islam Repub. Iran.

[39] Kassi E., Pervanidou P., Kaltsas G., Chrousos G. (2011). Metabolic syndrome: Definitions and controversies. BMC Med..

[40] Wells G.A., Shea B.J., O’Connell D., Peterson I.E. (2000). The Newcastle–Ottawa Scale (NOS) for Assessing Quality of Nonrandomised Studies in Meta-Analyses.

[41] Peterson J., Welch V., Losos M., Tugwell P.J. (2011). The NOS for Assessing Quality of Nonrandomised Studies in Meta-Analyses.

[42] Luchini C., Stubbs B., Solmi M., Veronese N. (2017). Advantages and limitations of the NOS. World J. Meta-Anal..

[43] Janem W.F., Scannapieco F.A., Sabharwal A., Tsompana M., Berman H.A., Haase E.M., Miecznikowski J.C., Mastrandrea I.D. (2017). Salivary inflammatory markers and microbiome in obese children with/without T2D. PLoS ONE.

[44] Sarhat E.R., Mohammed I.J., Hamad A.I. (2017). Biochemistry of serum and saliva in obese individuals with periodontitis: Case-control. J. Oral Dent. Res..

[45] Naidoo T., Konkol K., Biccard B., Dudose K., Mckune A.J. (2012). Elevated salivary CRP predicted by low fitness and overweight in African children. Cardiovasc. J. Afr..

[46] Agho E.T., Owotade F.J., Kolawole B.A., Oyetola E.O., Adedeji T.A. (2021). Salivary inflammatory biomarkers and HbA1c in T2DM. BMC Oral. Health.

[47] Labat C., Temmar M., Nagy E., Bean K., Brink C., Benetos A., Bäck M. (2013). Salivary inflammatory mediators and arterial stiffness/subclinical atherosclerosis. J. Hypertens..

[48] Mrag M., Kassab A., Omezzine A., Belkacem C.R., Ben F.I., Douki N., Kechrid C., Laouani A., Bouslema A., Ben A.F. (2020). Saliva diagnostic utility in T2D: Future standard method. J. Med. Biochem..

[49] Alqaderi H., Hegazi F., Al-Mulla F., Chiu C.J., Kantarci A., Al-Ozairi E., Abu-Farha M., Bin-Hasan S., Alsumait A., Abubaker J. (2022). Salivary biomarkers predicting obesity and intermediate hyperglycemia in adolescents. Front. Public Health.

[50] Selvaraju V., Babu J.R., Geetha T. (2019). Salivary CRP and obesity measures in children. Diabetes Metab. Syndr. Obes..

[51] Safabakhsh D., Jazaeri M., Abdolsamadi H., Abassi E., Farhadian M. (2022). Salivary IL-6, IL-8, CRP and antioxidants in obese vs normal weight. Rom. J. Intern. Med..

[52] Biziak D.A., Ammerpohl O., Schulz S.V., Wendt J., Steinacker J.M., Flechtner-Mors M. (2022). Pro-inflammatory and (epi-)genetic saliva markers in childhood obesity. Nutr. Metab. Cardiovasc. Dis..

[53] Goodson J.M., Kantarci A., Hartman M.L., Denis G.V., Stephens D., Hasturk H., Yaskell T., Vargas J., Wang X., Cugini M. (2014). Metabolic disease risk in children by salivary biomarker analysis. PLoS ONE.

[54] Tvarijonaviciute A., Martinez-Lozano N., Rios R., Marcilla de Teruel M.C., Garaulet M., Cerón J.J. (2020). Saliva for assessment of metabolic/inflammatory biomarkers in children. Clin. Nutr..

[55] Zambon M., Mandò C., Lissoni A., Anelli G.M., Novielli C., Cardellicchio M., Leone R., Monari M.N., Massari M., Cetin I. (2018). Inflammation/oxidative responses in pregnancies with obesity and periodontal disease. Reprod. Sci..

[56] Ebersole J.L., Kryscio R.J., Campbell C., Kinane D.F., McDevitt J., Christodoulides N., Floriano P.N., Miller C.S. (2017). Salivary/serum adiponectin and CRP in acute MI in relation to BMI and oral health. J. Periodontal Res..

[57] Ozmeric N., Elgun S., Kalfaoglu D., Pervane B., Sungur C., Ergüder I., Yavuz Y. (2024). Interaction between hypertension and periodontitis. Oral. Dis..

[58] Punyadeera C., Dimeski G., Kostner K., Beyerlein P., Cooper-White J. (2011). One-step homogeneous CRP assay for saliva. J. Immunol. Methods.

[59] McGeer P.L., Lee M., Kennedy K., McGeer E.G. (2020). Saliva diagnosis as a disease predictor. J. Clin. Med..

[60] Page M.J., McKenzie J.E., Bossut P.M., Boutron I., Hoffmann T.C., Mulrow C.D., Shamseer L., Tetzlaff J.M., Aki E.A., Brennan S.E. (2021). The PRISMA 2020 statement. BMJ.

[61] Bachtiar E., Bachtiar B.M., Kusumaningrum A., Sunarto H., Soeroso Y., Sulijaya B., Apriyanti E., Theodorea C.F., Pratomo I.P., Yudhistira Y. (2024). Utility of salivary CRP and IL-6 in COVID-19 with/without diabetes. F1000Research.

[62] Sugimoto M., Ota S., Kaneko M., Enomoto A., Soga T. (2020). Quantification of salivary charged metabolites using CE-TOF-MS. Bio-Protoc..

[63] Chukwuma D., Arowojolu M., Ankita J. (2024). Review of salivary biomarkers of periodontal disease. Ann. Ib. Postgrad. Med..

[64] Ajwani S., Mattila K.J., Närhi T.O., Tilvis R.S., Ainamo A. (2003). Oral health status, C-reactive protein and mortality—A 10 year follow-up study. Gerodontology.

[65] Akin F., Ayça B., Köse N., Celik O., Yilmaz Y., Akin M.N., Arinc H., Ozkok A., Covic A., Kanbay M. (2014). Serum vitamin D and C-reactive protein levels are independently associated with diastolic dysfunction. J. Investig. Med..

[66] Al Akl N.S., Khalifa O., Habibullah M., Arredouani A. (2024). Salivary α-amylase activity is associated with cardiometabolic and inflammatory biomarkers in overweight/obese, non-diabetic Qatari women. Front. Endocrinol..

[67] Al-Rawi N.H., Shahid A.M. (2017). Oxidative stress, antioxidants, and lipid profile in the serum and saliva of individuals with coronary heart disease: Is there a link with periodontal health?. Minerva Stomatol..

[68] Alqerban A., Asiri S.N., Alharbi F., Almalki A., Alqhtani N., Alenazi A., Robaian A., Samran A. (2023). Effect of ten different biomarkers in the gingival crevicular fluid of obese and non-obese undergoing fixed orthodontic treatment. Eur. Rev. Med. Pharmacol. Sci..

[69] Altingöz S.M., Kurgan Ş., Önder C., Serdar M.A., Ünlütürk U., Uyanik M., Başkal N., Tatakis D.N., Günhan M. (2021). Salivary and serum oxidative stress biomarkers and advanced glycation end products in periodontitis patients with or without diabetes: A cross-sectional study. J. Periodontol..

[70] Arshad M.K., Bin Mohamad Fathil M.F., Gopinath S.C., Ruslinda A.R., Md Nor M.N., Lam H.Y., Hashim U. (2016). Cardiac biomarkers: Invasive to non-invasive assessments. Curr. Med. Chem..

[71] Bahbah E.I., Noehammer C., Pulverer W., Jung M., Weinhaeusel A. (2021). Salivary biomarkers in cardiovascular disease: An insight into the current evidence. FEBS J..

[72] Bermúdez-Millán A., Wagner J.A., Feinn R.S., Segura-Pérez S., Damio G., Chhabra J., Pérez-Escamilla R. (2019). Inflammation and stress biomarkers mediate the association between household food insecurity and insulin resistance among Latinos with type 2 diabetes. J. Nutr..

[73] Brett B.E., Koko B.K., Doumbia H.O.Y., Koffi F.K., Assa S.E., Zahé K., Faye-Ketté H., Kati-Coulibaly S., Kort R., Sybesma W. (2021). Salivary biomarkers of stress and inflammation in first graders in Côte d’Ivoire: Effects of a probiotic food intervention. Psychoneuroendocrinology.

[74] Bulut A., Akca G., Keskin Aktan A., Akbulut K.G., Babül A. (2021). The significance of blood and salivary oxidative stress markers and chemerin in gestational diabetes mellitus. Taiwan J. Obstet. Gynecol..

[75] Caloian C.S., Șurlin P., Ciurea A., Pop D., Caloian B., Leucuța D.C., Țigu A.B., Rasperini G., Micu I.C., Stanomir A. (2024). Exploring periodontal conditions, salivary markers, and systemic inflammation in patients with cardiovascular diseases. Biomedicines.

[76] Can U., Buyukinan M., Guzelant A., Ugur A., Karaibrahimoglu A., Yabanciun S. (2016). Investigation of the inflammatory biomarkers of metabolic syndrome in adolescents. J. Pediatr. Endocrinol. Metab..

[77] Choromańska K., Choromańska B., Dąbrowska E., Bączek W., Myśliwiec P., Dadan J., Zalewska A. (2015). Saliva of obese patients—Is it different?. Postep. Hig. Med. Dosw..

[78] Chauhan A., Yadav S.S., Dwivedi P., Lal N., Usman K., Khattri S. (2016). Correlation of serum and salivary cytokines level with clinical parameters in metabolic syndrome with periodontitis. J. Clin. Lab. Anal..

[79] Christaki E.V., Pervanidou P., Papassotiriou I., Bastaki D., Valavani E., Mantzou A., Giannakakis G., Boschiero D., Chrousos G.P. (2022). Stress, inflammation and metabolic biomarkers are associated with body composition measures in lean, overweight, and obese children and adolescents. Children.

[80] Ramya D., Raghunath V., Krishna P.S., Kamal F., Aparna Latha H. (2025). Assessment of serum and salivary resistin levels in newly diagnosed type-II diabetes mellitus patients. J. Oral Maxillofac. Pathol..

[81] Davis S.L., Latimer M., Rice M. (2023). Biomarkers of stress and inflammation in children. Biol. Res. Nurs..

[82] De Jager W., Holzinger D., Rijkers G.T. (2013). Biomarkers in inflammatory childhood diseases. Mediat. Inflamm..

[83] Dekker R.L., Lennie T.A., Moser D.K., Miller C.S., Ebersole J.L., Chung M.L., Campbell C.L., Bailey A., Tovar E.G. (2017). Salivary biomarkers, oral inflammation, and functional status in patients with heart failure. Biol. Res. Nurs..

[84] Desai G.S., Mathews S.T. (2014). Saliva as a non-invasive diagnostic tool for inflammation and insulin-resistance. World J. Diabetes..

[85] Foley J.D., Sneed J.D., Steinhubl S.R., Kolasa J.R., Ebersole J.L., Lin Y., Kryscio R.J., McDevitt J.T., Campbell C.L., Miller C.S. (2012). Salivary biomarkers associated with myocardial necrosis: Results from an alcohol septal ablation model. Oral Surg. Oral Med. Oral Pathol. Oral Radiol..

[86] Janket S., Meurman J.H., Baird A.E., Qvarnström M., Nuutinen P., Ackerson L.K., Hong J., Muthukrishnan P., Van Dyke T.E. (2010). Salivary immunoglobulins and prevalent coronary artery disease. J. Dent. Res..

[87] Janket S.J., Baird A.E., Jones J.A., Jackson E.A., Surakka M., Tao W., Meurman J.H., Van Dyke T.E. (2014). Number of teeth, C-reactive protein, fibrinogen and cardiovascular mortality: A 15-year follow-up study in a Finnish cohort. J. Clin. Periodontol..

[88] Jerusha F.R., Raghunath V. (2023). Assessment of serum and salivary visfatin levels in newly diagnosed patients of type-II DM. J. Oral Maxillofac. Pathol..

[89] Jones B.L., Elwazeer S., Taylor Z.E. (2018). Salivary uric acid and C-reactive protein associations with hypertension. Dev. Psychobiol..

[90] Kalyani R.S., Raghunath V. (2020). Assessment of serum and salivary adiponectin levels in newly diagnosed Type II diabetes mellitus patients. J. Oral Maxillofac. Pathol..

[91] Klichowska-Palonka M., Załęska-Chromińska K., Bachanek T. (2015). Possibility of using saliva as a diagnostic test material in cardiovascular diseases. Wiad. Lek..

[92] Li P., He L., Chen Z.B., Luan Q.X. (2020). Biomarkers in metabolic syndrome patients with chronic periodontitis. Chin. J. Dent. Res..

[93] Meyer M.H., Hartmann M., Krause H.J., Blankenstein G., Mueller-Chorus B., Oster J., Miethe P., Keusgen M. (2007). CRP determination based on a novel magnetic biosensor. Biosens. Bioelectron..

[94] Nguyen T.T., Ngo L.Q., Promsudthi A., Surarit R. (2017). Salivary oxidative stress biomarkers in chronic periodontitis and acute coronary syndrome. Clin. Oral Investig..

[95] Plank A.C., Maschke J., Rohleder N., Fasching P.A., Beckmann M.W., Kornhuber J., Eichler A., Moll G.H., Kratz O. (2021). Comparison of C-reactive protein in dried blood spots and saliva of healthy adolescents. Front. Immunol..

[96] Qvarnstrom M., Janket S.J., Jones J.A., Jethwani K., Nuutinen P., Garcia R.I., Baird A.E., Van Dyke T.E., Meurman J.H. (2010). Association of salivary lysozyme and C-reactive protein with metabolic syndrome. J. Clin. Periodontol..

[97] Ridker P.M. (2007). C-reactive protein and the prediction of cardiovascular events among those at intermediate risk: Moving an inflammatory hypothesis toward consensus. J. Am. Coll. Cardiol..

[98] Selvaraju V., Babu J.R., Geetha T. (2022). Multiplexed measurements of salivary fetuin-A, insulin, and adiponectin in childhood obesity. Cytokine.

[99] Selvaraju V., Venkatapoorna C.M.K., Babu J.R., Geetha T. (2020). Salivary amylase gene copy number is associated with obesity and inflammatory markers in children. Diabetes Metab. Syndr. Obes..

[100] Shi P., Goodson J.M. (2019). Data mining approach identified salivary biomarkers. J. Obes..

[101] Malik A., Morya R.K., Saha S., Singh P.K., Bhadada S.K., Rana S.V. (2020). Oxidative stress and inflammatory markers in type 2 diabetic patients. Eur. J. Clin. Invest..

[102] Slavish D.C., Jones D.R., Smyth J.M., Engeland C.G., Song S., McCormick N.M., Graham-Engeland J.E. (2020). Positive and negative affect and salivary markers of inflammation among young adults. Int. J. Behav. Med..

[103] Speer H., D’Cunha N.M., Naumovski N., McKune A.J. (2021). Sex, age, BMI, and C-reactive protein impact the odds of developing hypertension—Findings based on data from the Health and Retirement Study (HRS). Am. J. Hypertens..

[104] Steigmann L., Maekawa S., Kauffmann F., Reiss J., Cornett A., Sugai J., Venegas J., Fan X., Xie Y., Giannobile W.V. (2022). Changes in salivary biomarkers associated with periodontitis and diabetic neuropathy in individuals with type 1 diabetes. Sci. Rep..

[105] Suzuki D., Yamada S.I., Sakurai A., Karasawa I., Kondo E., Sakai H., Tanaka H., Shimane T., Kurita H. (2020). Correlations between the properties of saliva and metabolic syndrome: A prospective observational study. Medicine.

[106] Timpson N.J., Lawlor D.A., Harbord R.M., Gaunt T.R., Day I.N., Palmer L.J., Hattersley A.T., Ebrahim S., Lowe G.D., Rumley A. (2005). C-reactive protein and its role in metabolic syndrome: Mendelian randomisation study. Lancet.

[107] Truba T.N., Doan J., Currie C.L., Copeland J.L. (2018). Short-term changes in daily movement behaviour influence salivary C-reactive protein in healthy women. Appl. Physiol. Nutr. Metab..

[108] Tsigos C., Stefanaki C., Lambrou G.I., Boschiero D., Chrousos G.P. (2015). Stress and inflammatory biomarkers and symptoms are associated with bioimpedance measures. Eur. J. Clin. Investig..

[109] Van Leeuwen W.M., Lehto M., Karisola P., Lindholm H., Luukkonen R., Sallinen M., Härmä M., Porkka-Heiskanen T., Alenius H. (2009). Sleep restriction increases the risk of developing cardiovascular diseases by augmenting proinflammatory responses through IL-17 and CRP. PLoS ONE.

[110] Varma S., Thomas B., Subrahmanyam K., Duarte K., Alsaegh M.A., Gopinath D., Kuriadom S.T., Narayanan J., Desai V.B., Khair A.M.B. (2024). Salivary levels of inflammatory and anti-inflammatory biomarkers in periodontitis patients with and without acute myocardial infarction: Implications for cardiovascular risk assessment. Front. Oral Health.

[111] Wetterö J., Von Löhneysen S., Cobar F., Kristenson M., Garvin P., Sjöwall C. (2020). Pronounced diurnal pattern of salivary C-reactive protein (CRP) with modest associations to circulating CRP levels. Front. Immunol..

[112] Yin X., Subramanian S., Hwang S.J., O’Donnell C.J., Fox C.S., Courchesne P., Muntendam P., Gordon N., Adourian A., Juhasz P. (2014). Protein biomarkers of new-onset cardiovascular disease: Prospective study from the systems approach to biomarker research in cardiovascular disease initiative. Arterioscler. Thromb. Vasc. Biol..

