# Supplementary material for: C-Reactive Protein in Saliva as a Non-Invasive Marker of Metabolic Syndrome: A Systematic Review and Meta-Analysis
Source: Life (Basel). 2026 Mar 2;16(3):403. doi: 10.3390/life16030403 (PMC13028261; doi:10.3390/life16030403)
Supplement: Supplementary file 1 [file life-16-00403-s001.zip › life-4057624-supplementary.pdf]

Supplementary File S1. Comprehensive Search Strategy Applied Across All Databases

((saliva OR salivary) AND (CRP OR "c reactive protein" OR "C-Reactive Protein"[Mesh]) AND ("metabolic syndrome" OR "Metabolic Syndrome"[Mesh] OR "met S" OR obesity OR BMI OR "body mass index" OR "waist circumference" OR diabetes OR hyperglycemia OR hbA1C OR "high glucose" OR prediabetes OR hypertension OR "high blood pressure" OR "cardiovascular disease" OR "Cardiovascular Diseases"[Mesh] OR "CAD" OR "coronary artery disease" OR "heart disease" OR "high cholesterol" OR "high LDL" OR "low HDL" OR hypercholesterolemia))

Supplementary File S2. Excluded Articles with Reasons for Exclusion

66 excluded full text articles

Ajwani, S., et al., Oral health status, C-reactive protein and mortality--a 10 year follow-up study. Gerodontology, 2003. 20(1): p. 32-40.

Metabolic syndrome not assessed

Akin, F., et al., Serum Vitamin D and C- Reactive Protein Levels Are Independently Associated With Diastolic Dysfunction. Journal of Investigative Medicine, 2014. 62(1): p. 43-48.

Salivary C-reactive protein not assessed

Al Akl, N.S., et al., Salivary Œ±-amylase activity is associated with cardiometabolic and inflammatory biomarkers in overweight/obese, non-diabetic Qatari women. Front Endocrinol (Lausanne), 2024. 15: p. 1348853.

Salivary C-reactive protein not assessed

Al-Rawi, N.H. and A.M. Shahid, Oxidative stress, antioxidants, and lipid profile in the serum and saliva of individuals with coronary heart disease: is there a link with periodontal health? Minerva Stomatol, 2017. 66(5): p. 212-225.

Abstract without full-text publication

Alqerban, A., et al., Effect of ten different biomarkers in the gingival crevicular fluid of obese and non-obese undergoing fixed orthodontic treatment. European Review for Medical and Pharmacological Sciences, 2023. 27(5): p. 1722-1728.

Salivary C-reactive protein not assessed

Altingoz, S.M., et al., Salivary and serum oxidative stress biomarkers and advanced glycation end products in periodontitis patients with or without diabetes: A cross-sectional study. Journal of Periodontology, 2021. 92(9): p. 1274-1285.

Wrong comparator

Ansar, W. and S. Ghosh, C-reactive protein and the biology of disease. Immunologic research, 2013. 56: p. 131-142.

Inappropriate study design

Arshad, M.K., et al., Cardiac Biomarkers: Invasive to Non-invasive Assessments. Curr Med Chem, 2016. 23(37): p. 4270-4284.

Inappropriate study design

Bahbah, E.I., et al., Salivary biomarkers in cardiovascular disease: An insight into the current evidence. Febs j, 2021. 288(22): p. 6392-6405.

Inappropriate study design

Bermudez-Millan, A., et al., Inflammation and Stress Biomarkers Mediate the Association between Household Food Insecurity and Insulin Resistance among Latinos with Type 2 Diabetes. Journal of Nutrition, 2019. 149(6): p. 982-988.
Wrong outcome

Brett, B.E., et al., Salivary biomarkers of stress and inflammation in first graders in Co<SIC>te d,Ä≤Ivoire: Effects of a probiotic food intervention. Psychoneuroendocrinology, 2021. 129.
Wrong outcome

Bulut, A., et al., The significance of blood and salivary oxidative stress markers and chemerin in gestational diabetes mellitus. Taiwanese Journal of Obstetrics & Gynecology, 2021. 60(4): p. 695-699.

Salivary C-reactive protein not assessed

Caloian, C.S., et al., Exploring Periodontal Conditions, Salivary Markers, and Systemic Inflammation in Patients with Cardiovascular Diseases. Biomedicines, 2024. 12(6)

Salivary C-reactive protein not assessed

Cameron, A.J., J.E. Shaw, and P.Z. Zimmet, The metabolic syndrome: prevalence in worldwide populations. Endocrinology and Metabolism Clinics, 2004. 33(2): p. 351-375.

Inappropriate study design

Can, U., et al., Investigation of the inflammatory biomarkers of metabolic syndrome in adolescents. Journal of Pediatric Endocrinology and Metabolism, 2016. 29(11): p. 1277-1283.

Salivary C-reactive protein not assessed

Castagnola, M., et al., Potential applications of human saliva as diagnostic fluid. Acta Otorhinolaryngologica Italica, 2011. 31(6): p. 347.

Inappropriate study design

Choroma≈Ñska, K., et al., Saliva of obese patients - is it different? Postepy Hig Med Dosw (Online), 2015. 69: p. 1190-5.

Inappropriate study design

Chauhan, A., et al., *Correlation of Serum and Salivary Cytokines Level With Clinical Parameters in Metabolic Syndrome With Periodontitis.*Journal of Clinical Laboratory Analysis, 2016. 30(5): p. 649-655.

Salivary C-reactive protein not assessed

Christaki, E.V., et al., Stress, Inflammation and Metabolic Biomarkers Are Associated with Body Composition Measures in Lean, Overweight, and Obese Children and Adolescents. Children (Basel), 2022. 9(2).

Salivary C-reactive protein not assessed

Ctri, To study the relationship of salivary hs-CRP with the regular cardiovascular risk factors and HbA1c in prediabetic patients. https://trialsearch.who.int/Trial2.aspx?TrialID=CTRI/2024/03/064537, 2024.

Wrong comparator

Davis, S.L., M. Latimer, and M. Rice, Biomarkers of Stress and Inflammation in Children. Biol Res Nurs, 2023. 25(4): p. 559-570.

Inappropriate study design

de Jager, W., D. Holzinger, and G.T. Rijkers, Biomarkers in Inflammatory Childhood Diseases. Mediators of Inflammation, 2013. 2013.

Inappropriate study design

Dekker, R.L., et al., Salivary Biomarkers, Oral Inflammation, and Functional Status in Patients With Heart Failure. Biol Res Nurs, 2017. 19(2): p. 153-161.

Inadequate comparator

Desai, G.S. and S.T. Mathews, Saliva as a non-invasive diagnostic tool for inflammation and insulin-resistance. World J Diabetes, 2014. 5(6): p. 730-8.

Inappropriate study design

24. Dezayee, Z.M. and M.S. Al-Nimer, Saliva C-reactive protein as a biomarker of metabolic syndrome in diabetic patients. Indian J Dent Res, 2016. 27(4): p. 388-391.

Insufficient data for meta-analysis

Foley, J.D., 3rd, et al., Salivary biomarkers associated with myocardial necrosis: results from an alcohol septal ablation model. Oral Surg Oral Med Oral Pathol Oral Radiol, 2012. 114(5): p. 616-23.

Inappropriate study design

Hotamisligil, G.S., Inflammation and metabolic disorders. Nature, 2006. 444(7121): p. 860-867.

Inappropriate study design

Janket, S., et al., Salivary immunoglobulins and prevalent coronary artery disease. J Dent Res, 2010. 89(4): p. 389-94.

Insufficient data for meta-analysis

Janket, S.-J., et al., Number of teeth, C-reactive protein, fibrinogen and cardiovascular mortality: a 15-year follow-up study in a Finnish cohort. Journal of Clinical Periodontology, 2014. 41(2): p. 131-140.

Insufficient data for meta-analysis

Javaid, M.A., et al., Saliva as a diagnostic tool for oral and systemic diseases. Journal of oral biology and craniofacial research, 2016. 6(1): p. 67-76.

Inappropriate study design

30. Jerusha, F.R. and V. Raghunath, Assessment of serum and salivary visfatin levels in newly diagnosed patients of type-II DM. J Oral Maxillofac Pathol, 2023. 27(4): p. 663-667.

Salivary C-reactive protein not assessed

31. Jones, B.L., S. Elwazeer, and Z.E. Taylor, Salivary uric acid and C-reactive protein associations with hypertension in Midwestern Latino preadolescents and their parents. Dev Psychobiol, 2018. 60(1): p. 104-110.

Insufficient data for meta-analysis

32. Kalyani, R.S. and V. Raghunath, Assessment of serum and salivary adiponectin levels in newly diagnosed Type II diabetes mellitus patients. J Oral Maxillofac Pathol, 2020. 24(2): p. 245-250.

Salivary C-reactive protein not assessed

33. Klichowska-Palonka, M., K. Za≈Çƒôska-Chromi≈Ñska, and T. Bachanek, [Possibility of using saliva as a diagnostic test material in cardiovascular diseases]. Wiad Lek, 2015. 68(3 pt 2): p. 354-357.

Inappropriate study design

34. Li, P., et al., Biomarkers in Metabolic Syndrome Patients with Chronic Periodontitis. Chin J Dent Res, 2020. 23(3): p. 191-197.

Salivary C-reactive protein not assessed

35. Malamud, D. and I.R. Rodriguez-Chavez, Saliva as a diagnostic fluid. Dental Clinics of North America, 2011. 55(1): p. 159.

Inappropriate study design

36. Malon, R.S., et al., Saliva‚Äêbased biosensors: noninvasive monitoring tool for clinical diagnostics. BioMed research international, 2014. 2014(1): p. 962903.

Inappropriate study design

37. Meyer, M.H.F., et al., CRP determination based on a novel magnetic biosensor. Biosensors & Bioelectronics, 2007. 22(6): p. 973-979.

Salivary C-reactive protein not assessed

38. Miller, C.S., et al., Current developments in salivary diagnostics. Biomarkers in medicine, 2010. 4(1): p. 171-189.

Inappropriate study design

39. Nguyen, T.T., et al., Salivary oxidative stress biomarkers in chronic periodontitis and acute coronary syndrome. Clin Oral Investig, 2017. 21(7): p. 2345-2353.

Salivary C-reactive protein not assessed

40. Ouellet-Morin, I., et al., Validation of a high-sensitivity assay for C-reactive protein in human saliva. Brain, behavior, and immunity, 2011. 25(4): p. 640-646.

Metabolic syndrome not assessed

41. Out, D., et al., Assessing salivary C-reactive protein: longitudinal associations with systemic inflammation and cardiovascular disease risk in women exposed to intimate partner violence. Brain, behavior, and immunity, 2012. 26(4): p. 543-551.

Insufficient data for meta-analysis

42. Pearson, T.A., et al., Markers of inflammation and cardiovascular disease: application to clinical and public health practice: a statement for healthcare professionals from the Centers for Disease Control and Prevention and the American Heart Association. circulation, 2003. 107(3): p. 499-511.

Salivary C-reactive protein not assessed

43. Pfaffe, T., et al., Diagnostic potential of saliva: current state and future applications. Clinical chemistry, 2011. 57(5): p. 675-687.

Inappropriate study design

44. Plank, A.C., et al., Comparison of C-Reactive Protein in Dried Blood Spots and Saliva of Healthy Adolescents. Front Immunol, 2021. 12: p. 795580.

Insufficient data for meta-analysis

45. Pradhan, A.D., et al., C-reactive protein, interleukin 6, and risk of developing type 2 diabetes mellitus. jama, 2001. 286(3): p. 327-334.

Salivary C-reactive protein not assessed

46. Qvarnstrom, M., et al., Association of salivary lysozyme and C-reactive protein with metabolic syndrome. J Clin Periodontol, 2010. 37(9): p. 805-11.

Salivary C-reactive protein not assessed

48. Ridker, P.M., et al., C-reactive protein, the metabolic syndrome, and risk of incident cardiovascular events: an 8-year follow-up of 14 719 initially healthy American women. Circulation, 2003. 107(3): p. 391-397.

Salivary C-reactive protein not assessed

49. Ridker, P.M., et al., C-reactive protein and other markers of inflammation in the prediction of cardiovascular disease in women. New England journal of medicine, 2000. 342(12): p. 836-843.
Salivary C-reactive protein not assessed

50. Saklayen, M.G., The Global Epidemic of the Metabolic Syndrome. Current Hypertension Reports, 2018. 20(2): p. 12.

Inappropriate study design

51. Samson, S.L. and A.J. Garber, Metabolic syndrome. Endocrinology and Metabolism Clinics, 2014. 43(1): p. 1-23.

Inappropriate study design

52. Selvaraju, V., J.R. Babu, and T. Geetha, Multiplexed measurements of salivary fetuin-A, insulin, and adiponectin as potential non-invasive biomarkers in childhood obesity. Cytokine, 2022. 153.
Salivary C-reactive protein not assessed

53. Selvaraju, V., et al., Salivary Amylase Gene Copy Number Is Associated with the Obesity and Inflammatory Markers in Children. Diabetes Metab Syndr Obes, 2020. 13: p. 1695-1701.
Salivary C-reactive protein not assessed

54. Shi, P. and J.M. Goodson, A Data Mining Approach Identified Salivary Biomarkers That Discriminate between Two Obesity Measures. J Obes, 2019. 2019: p. 9570218.

Insufficient data for meta-analysis

55. Siddiqui, A., et al., Association of oxidative stress and inflammatory markers with chronic stress in patients with newly diagnosed type 2 diabetes. Diabetes Metab Res Rev, 2019. 35(5): p. e3147.
Salivary C-reactive protein not assessed

56. Slavish, D.C., et al., Positive and Negative Affect and Salivary Markers of Inflammation Among Young Adults. Int J Behav Med, 2020. 27(3): p. 282-293.
Metabolic syndrome not assessed

57. Speer, H., et al., Sex, Age, BMI, and C-Reactive Protein Impact the Odds of Developing Hypertension-Findings Based on Data From the Health and Retirement Study (HRS). Am J Hypertens, 2021. 34(10): p. 1057-1063.

Insufficient data for meta-analysis

58. Steigmann, L., et al., Changes in salivary biomarkers associated with periodontitis and diabetic neuropathy in individuals with type 1 diabetes. Scientific Reports, 2022. 12(1).

Insufficient data for meta-analysis

59. Suzuki, D., et al., Correlations between the properties of saliva and metabolic syndrome A prospective observational study. Medicine, 2020. 99(51).

C-reactive protein not assessed

60. Timpson, N.J., et al., C-reactive protein and its role in metabolic syndrome: mendelian randomisation study. The Lancet, 2005. 366(9501): p. 1954-1959.

Salivary C-reactive protein not assessed

61. Truba, T.N., et al., Short-term changes in daily movement behaviour influence salivary C-reactive protein in healthy women. Applied Physiology Nutrition and Metabolism, 2018. 43(8): p. 854-856.

Metabolic syndrome not assessed

62. Tsigos, C., et al., Stress and inflammatory biomarkers and symptoms are associated with bioimpedance measures. Eur J Clin Invest, 2015. 45(2): p. 126-34.

Metabolic syndrome not assessed

63. van Leeuwen, W.M.A., et al., Sleep Restriction Increases the Risk of Developing Cardiovascular Diseases by Augmenting Proinflammatory Responses through IL-17 and CRP. Plos One, 2009. 4(2).

Salivary C-reactive protein not assessed

64. Varma, S., et al., Salivary levels of inflammatory and anti-inflammatory biomarkers in periodontitis patients with and without acute myocardial infarction: implications for cardiovascular risk assessment. Front Oral Health, 2024. 5: p. 1332980.

Wrong comparator

65. Wetter√∂, J., et al., Pronounced Diurnal Pattern of Salivary C-Reactive Protein (CRP) With Modest Associations to Circulating CRP Levels. Front Immunol, 2020. 11: p. 607166.

Metabolic syndrome not assessed

66. Yin, X., et al., Protein Biomarkers of New-Onset Cardiovascular Disease Prospective Study From the Systems Approach to Biomarker Research in Cardiovascular Disease Initiative. Arteriosclerosis Thrombosis and Vascular Biology, 2014. 34(4): p. 939-945.

Salivary C-reactive protein not assessed
